# Is there a role for chemotherapy in prostate cancer?

**DOI:** 10.1038/sj.bjc.6601850

**Published:** 2004-04-27

**Authors:** C M Canil, I F Tannock

**Affiliations:** 1 Department of Medical Oncology and Hematology, Princess Margaret Hospital, University Health Network, 610 University Avenue, Toronto, Ontario, Canada M5G 2M9

**Keywords:** prostate cancer, chemotherapy, adjuvant, palliative, mitoxantrone, taxanes

## Abstract

There is evidence from randomised-controlled trials that patients with symptomatic hormone-refractory prostate cancer may experience palliative benefit from chemotherapy with mitoxantrone and prednisone. This treatment is well tolerated, even by elderly patients, although the cumulative dose of mitoxantrone is limited by cardiotoxicity. Treatment with docetaxel or paclitaxel, with or without estramustine, appears to convey higher rates of prostate-specific antigen response in phase II trials, but is more toxic. Large phase III trials comparing docetaxel with mitoxantrone have completed accrual. There is no role for chemotherapy in earlier stages of disease except in the context of a well-designed clinical trial.

Chemotherapy has not been shown to alter survival in prostate cancer. However, in recent years, clinical trials have provided evidence for a palliative role of chemotherapy in patients with symptomatic hormone-refractory disease. In addition, new chemotherapeutic agents have shown promising results in phase II studies leading to enthusiasm for challenging the belief that chemotherapy does not alter the natural history of prostate cancer. In this article, we will review the role of chemotherapy during various stages of prostate cancer, using hypothetical case reports to illustrate our evidence-based discussion.

## POSSIBLE ROLE OF CHEMOTHERAPY FOR LOCALISED PROSTATE CANCER

Mr Smith is a healthy 55-year-old male with a strong family history of prostate cancer. A prostate-specific antigen (PSA) serum test was included as part of his annual medical examination. The PSA value was elevated at 22 *μ*g l^−1^ (normal: <4 *μ*g l^−1^). Rectal ultrasound revealed a bulky nodular prostate staged clinically as T2b and subsequent biopsy showed a Gleason score of 4+4/10. Bone and CT scans of the abdomen and pelvis were negative for metastatic disease. After discussion with an urologist and a radiation oncologist, Mr Smith decided to proceed with radical prostatectomy. After searching the internet, he asks his doctors if use of hormones and/or chemotherapy would decrease the risk of recurrent disease.

Clinical and pathologic features including the serum level of PSA, Gleason score and clinical stage can be used to estimate the probability that a patient will develop recurrent disease after radical local therapy with surgery or radiation. These factors have been incorporated into nomograms (Partin *et al*, 1997; Kattan *et al*, 1998). Using the Partin nomograms, Mr Smith has a risk of capsular penetration of about 28%, seminal vesicle involvement of 33% and lymph node involvement of 36%. He also has a substantial probability of positive margins after radical prostatectomy. According to the Kattan nomogram, Mr Smith has only a 20% (∓10%) probability of remaining free of disease at 5 years following radical prostatectomy. High rates of relapse are due both to local failure and to systemic failure secondary to the presence of micrometastatic disease. Thus, Mr Smith's question about adjuvant therapy is appropriate.

The goals of adjuvant therapy are to destroy residual local and micrometastatic disease, while tumour burden is low and patient performance status is good with the intent of curing the patient of the cancer. When adjuvant therapy is given before the primary treatment, it is referred to as neoadjuvant therapy. Neoadjuvant treatment might also decrease the incidence of positive margins in patients undergoing radical prostatectomy and thereby increase local control.

### Adjuvant hormonal therapy

When systemic therapy is considered in the management of patients with prostate cancer, hormonal therapy is, in general, much more effective than cytotoxic chemotherapy. Androgen ablation (by orchidectomy or luteinising hormone-releasing hormone (LHRH) agonist, with or without a peripheral antiandrogen), is effective in providing a reduction in tumour volume, fall in PSA and/or relief of clinical symptoms in about 80% of patients with advanced disease, and the average duration of response is 1–2 years.

For patients with localised T2 prostate cancer, androgen ablation given 3 months before surgery has been shown to reduce the stage of disease at surgery and to decrease the rate of positive margins. However, no differences in biochemical or local recurrence rates have been demonstrated (Goldenberg *et al*, 1996; Klotz *et al*, 1999; Soloway *et al*, 2002). Criticisms of these randomised-controlled trials include the short duration of neoadjuvant treatment, lack of statistical power and lack of long-term follow-up. Two studies have compared adjuvant treatment with an LHRH agonist to observation after radical prostatectomy. In the Eastern Cooperative Oncology Group (ECOG) study, 98 patients with node-positive disease found at radical prostatectomy were randomised to immediate antiandrogen therapy or observation until disease progression. After a median follow-up of 7 years, disease progression was reduced and survival was improved (Messing *et al*, 1999). In the second study published only in abstract form, 201 patients with pT3 disease were randomised to adjuvant goserelin or observation. Patients receiving hormonal therapy showed an improvement in disease-free survival at a median follow-up of 5 years (Prayer-Galetti *et al*, 2000).

There have been several large, well-designed trials of adjuvant antiandrogen therapy used with radiation therapy (Lawton *et al*, 2001; Bolla *et al*, 2002). Within these studies, the duration of androgen ablation varied from 4 months to indefinite, and hormonal therapy was initiated at the start of the course of radiation. Most studies have reported significant improvements in progression-free survival with the addition of adjuvant hormonal therapy. A meta-analysis of five randomised clinical trials involving >2700 patients undertaken by the Radiation Therapy Oncology Group (RTOG) analysed the impact of androgen suppression on the disease-specific and overall survival in patients treated with radiation therapy (Roach *et al*, 2000). Patients with high-risk disease, based on clinical stage and Gleason score, were noted to have about 20% higher survival at 8 years with the addition of long-term hormonal therapy. Patients with intermediate risk (bulky or T3 disease) appeared to have better disease-specific survival at 8 years with the addition of only 4 months of goserelin and flutamide. These results suggest that subgroups of patients can be identified who either do not benefit from the use of hormonal therapy, benefit from short-term hormonal therapy or who benefit only from long-term hormonal therapy.

### Adjuvant chemotherapy

Few studies have investigated the use of chemotherapy as adjuvant to local therapy for patients with prostate cancer. Most of these trials have used a combination of androgen ablation and chemotherapy. While it is logical to include hormonal therapy because of its greater effectiveness, this may lead to a higher proportion of noncycling cells and decreased effectiveness of cycle-active drugs. For patients with breast cancer, a large clinical trial has shown that sequential treatment with chemotherapy and tamoxifen gives a superior outcome to concurrent treatment and this may be relevant to treatment of prostate cancer (Albain *et al*, 2002).

Experience with neoadjuvant chemotherapy prior to radical prostatectomy is summarised in Table 1

**Table 1:** Summary of clinical trials of neoadjuvant chemotherapy followed by radical prostatectomy

**Author**	**Definition of high-risk group**	**Chemotherapy+androgen ablation**	***N***	**End points/conclusions**
Pettaway *et al* (2000)	Clinical stage T1–2, Gleason score ⩾8 or T2B-2c, Gleason score 7, PSA >10 ng ml^−1^ or clinical stage T3	12 w of ketoconazole and doxorubicin alternating with vinblastine, estramustine (KAVE)	33	Surgery feasible (30 had RP) No PT0 10/30 (33%) organ confined disease 20/30 (70%) extracapsular extension 11/30 (37%) positive lymph nodes 5/30 (17%) positive surgical margins
				
Clark *et al* (2001)	Clinical stage T2b or T3, PSA, PSA ⩾15 ng ml^−1^ or Gleason score⩾8	Three cycles of estramustine, and oral etoposide q 4 w	16	Surgery feasible 5/16 (31%) organ confined disease
				
Ko *et al* (2002)	Clinical stage T3 or PSA ⩾20 ng ml^−1^ or Gleason score ⩾8 or clinical stage T2 with MRI evidence of seminal vesicle involvement or Gleason score 4+3 with ⩾5 positive core biopsies	Four cycles of estramustine and docetaxel (70 mg m^−2^) i.v. q 3 w	12	Surgery feasible 1/12 (8%) lymph node positive 3/12 (25%) positive surgical margins
				
Hussain *et al* (2003)	Clinical stage ⩾T2b or PSA ⩾15 ng ml^−1^ or Gleason score ⩾8	Three to six cycles of estramustine and docetaxel (70 mg m^−2^) q 3 w	21	10 patients had RP (other 11 had RT as definitive local therapy) 3/10 (30%) positive surgical margins

MRI=Magnetic resonance imaging; *N*=number of patients; PSA=prostate-specific antigen; q=every; RP=radical prostatectomy; w=week; RT=radiation therapy.

. Pettaway *et al* (2000) enrolled 33 patients with high-risk localised prostate cancer to 12 weeks of ketoconazole (a general inhibitor of steroid synthesis) and doxorubicin alternating with vinblastine, estramustine (KAVE) and androgen ablation followed by prostatectomy. The primary objectives were to induce a 20% rate of complete pathological response in the prostatectomy specimen and to assess the impact on surgical morbidity. Of the 30 patients who completed systemic therapy and underwent radical prostatectomy, there was no detectable increase in the incidence of complications (33%) as compared to historical controls. However, no patient was downstaged to pT0 by the systemic treatment. Clark *et al* treated 18 patients with locally advanced prostate cancer with three cycles of estramustine and etoposide followed by radical prostatectomy. At surgery, there was residual disease in all patients, although the rate of organ-confined disease (31%) was a little higher than predicted by the Partin nomograms, and only two patients had positive margins (Clark *et al*, 2001). In addition, all patients achieved an undetectable PSA level postoperatively and at a median follow-up of 14 months. High rates of PSA response to docetaxel in hormone-refractory prostate cancer (see below) have led to several ongoing neoadjuvant studies of docetaxel with and without androgen ablation prior to radical prostatectomy. (Dreicer and Klein, 2001; Oh *et al*, 2001; Ko *et al*, 2002; Hussain *et al*, 2003).

The 10-year follow-up data are available for 2 three-arm randomised clinical trials conducted by the National Prostate Cancer Project of adjuvant chemotherapy. For patients with localised disease but at high risk of relapse, patients were randomised to cyclophosphamide 1 g m^−2^ every 3 weeks for 2 years, estramustine 600 mg m^−2^ orally daily for 2 years or observation, after the investigators choice of definitive therapy with either surgery (Protocol 900 – 170 evaluable patients) or radiation (Protocol 1000 – 233 evaluable patients) (Schmidt *et al*, 1996). No benefit was seen in overall survival, but a significantly longer median progression-free survival was noted in subgroups of stage C (surgery protocol) or grade 3 tumours (both radiation and surgery protocols) following treatment with estramustine. However, one must interpret these conclusions with some caution, as the analysis by subgroup was not planned *a priori*. It is unclear whether this effect (if real) is related to the estrogenic (estradiol) and/or alkylating (nitrogen mustard) properties of estramustine.

Several studies are underway to assess the adjuvant role of chemotherapy with definitive localised treatment (Gilligan and Kantoff, 2002). These include the Southwest Oncology Group (SWOG) 9921 study, in which patients are randomised to androgen deprivation plus mitoxantrone and prednisone or to androgen deprivation alone following prostatectoctomy. The RTOG 9902 study is comparing combined androgen blockade with paclitaxel, etoposide and estramustine chemotherapy after radiation. A third study will evaluate weekly docetaxel (without androgen ablation) in high-risk patients postprostatectomy.

Based on the above studies, there is no role for neoadjuvant or adjuvant chemotherapy combined with either surgery or radiation for patients with high-risk localised prostate cancer, other than in the setting of a well-designed clinical trial. Mr Smith should not receive chemotherapy for management of his disease.

## CHEMOTHERAPY IN ASYMPTOMATIC PATIENTS WITH HORMONE-REFRACTORY PROSTATE CANCER

Mr Smith has a radical prostatectomy. His PSA falls to undetectable levels, but within 2 years he has biochemical relapse and he receives local radiation and is started on hormonal therapy. His PSA falls again to undetectable levels, but 3 years later, his PSA increases and continues to rise despite continued treatment with an LHRH agonist and addition and withdrawal of bicalutamide, a peripheral antiandrogen. Mr Smith now has hormone-refractory disease and his most recent PSA is 35 *μ*g l^−1^, with a PSA doubling time of about 3 months. Bone scan shows several metastatic lesions, but there is no lymphadenopathy seen on abdominopelvic CT scan. Mr Smith feels well other than distress from knowing that his PSA is rising. Would Mr Smith benefit from chemotherapy?

In the past, the results of chemotherapy in patients with prostate cancer have been disappointing. For example, Yagoda and Petrylak (1993) reviewed 26 trials of chemotherapy for hormone-refractory prostate cancer and found a tumour response rate (using a variety of criteria) of less than 10%. Measuring tumour response in clinical trials of hormone-refractory prostate cancer has been problematic as few patients have measurable soft-tissue disease and the majority of patients have metastases to bone, a location that is difficult to assess for defining tumour response.

Assessing response of serum PSA can be useful in phase II trials that evaluate antitumour activity of new drugs. The Prostate-Specific Antigen Working Group standardised the definition of PSA response to facilitate comparison between phase II studies (Bubley *et al*, 1999). This group suggested that PSA response require a decline of at least 50% in serum PSA that must be confirmed by a second PSA value 4 or more weeks later. However, a decline in serum levels of PSA does not necessarily predict for clinical benefit, other than in relieving patient distress due to knowledge of PSA levels. In addition, one must be wary of comparing phase II studies since outcomes, such as PSA response, may be as dependent on selection of patients as on the agent under investigation.

No clinical trial has thus far demonstrated a survival benefit as a result of using chemotherapy in patients with prostate cancer. As hormone-refractory prostate cancer is not curable, the goals of therapy are to make patients live longer or live better. Appropriate end points for phase III studies are therefore overall survival and symptom control (or quality of life).

Patients with hormone-refractory prostate cancer are a heterogeneous group with respect to age, symptoms, comorbidities and presence or absence of metastatic disease. Few studies have focused exclusively on patients with asymptomatic hormone-refractory prostate cancer.

Mitoxantrone is an anthracycline cytotoxic drug, which interferes with DNA replication. As will be discussed later, mitoxantrone and prednisone is used for palliation of symptoms secondary to advanced cancer. However, Berry *et al* (2002) randomised 120 men with asymptomatic, hormone-refractory, progressive prostate cancer to mitoxantrone plus prednisone *vs* prednisone alone (Berry *et al*, 2002). The primary end point of this study was time to treatment failure, defined by the interval between the date of starting treatment and occurrence of progressive disease (increase in size of measurable masses, new soft-tissue lesions or increasing bone lesions). Secondary end points were PSA response, tumour response, duration of response and survival. Time to treatment failure was 8 months in the mitoxantrone and prednisone group compared to 4 months in the prednisone alone group, but there was no significant difference in overall survival. However, patients randomised to prednisone alone are likely to have received mitoxantrone or other chemotherapy after progression of disease. Unfortunately, this study did not determine whether early intervention with chemotherapy in hormone-refractory disease delayed the onset of symptoms, as there was no assessment of symptoms or quality of life data other than assessment of toxicity secondary to chemotherapy, which was minimal.

In summary, there are no data to indicate that an asymptomatic patient, such as Mr Smith in Scenario 2, would benefit from chemotherapy. Most trials of patients with hormone-refractory disease have included a mixture of symptomatic and asymptomatic patients.

## CHEMOTHERAPY IN SYMPTOMATIC PATIENTS WITH HORMONE-REFRACTORY PROSTATE CANCER

Mr Smith decides to enrol in a clinical trial of a biological agent. Unfortunately, his PSA continues to rise and he now begins to experience symptoms of pain in his lower back. MRI shows metastatic disease in the lumbar spine, but no evidence of cord compression. He is started on analgesics and bisphosphonates and given local radiotherapy to his lumbar spine. His lower back pain is relieved; however, he still complains of generalised aches in his bones and is very tired. Is there now a role for chemotherapy?

The median survival of patients with symptomatic hormone-refractory prostate cancer is 9–12 months. In later stages of disease, patients with prostate cancer often experience fatigue, anorexia, weight loss and pain because of bone metastases. Radiation therapy can be used to relieve localised bone pain, but metastases are usually widespread in bone, so that pain is often experienced in multiple sites. Analgesics and bisphosphonates may help with diffuse bone pain, but do not relieve the constitutional symptoms. Over time patients become increasingly weak and frail. The following agents might be considered in this situation.

### GLUCOCORTICOIDS

Glucocorticoids, primarily dexamethasone and prednisone, can play a useful role in the management of hormone-refractory prostate cancer. In symptomatic patients, steroids have been used to improve appetite, weight loss, fatigue and pain secondary to bone metastates. In addition, steroids are also important in the prevention of potential side effects of chemotherapy such as nausea, vomiting and allergic reactions.

Small phase II studies of glucocorticoid therapy in patients with progressive prostate cancer after medical or surgical castration have been reported to show PSA response rates of 40–60% (Storlie *et al*, 1995; Saika *et al*, 2001; Morioka *et al*, 2002; Akakura *et al*, 2003). The mechanism of action of glucocorticoids is not clearly understood and is thought to involve suppression of adrenal androgens (Tannock *et al*, 1989). However, these studies are difficult to interpret and compare as patient populations are highly selected, and are heterogeneous in the intensity of previous and concomitant therapies (Sartor *et al*, 1998). Many patients involved in these studies were not truly ‘hormone refractory’ as they did neither receive adequate treatment with peripheral antiandrogen therapy nor a trial of antiandrogen withdrawal prior to the initiation of glucocorticoid therapy. Better assessment of the role of glutocorticoids alone can be obtained from the larger randomised phase III trials that included prednisone or hydrocortisone alone as a control arm. In these studies, the rate of PSA response was in the range 16–24%, and generally of short duration (Tannock *et al*, 1996; Kantoff *et al*, 1999; Small *et al*, 2000b; Berry *et al*, 2002). In symptomatic patients, prednisone or hydrocortisone treatment was inferior in improving pain and other symptoms to arms that included mitoxantrone and steroid. While higher doses of the more potent glucocorticoid, dexamethasone might be more effective, another small study found no responses to 3 weeks of intensive treatment with dexamethasone (Weitzman *et al*, 2000).

A trial of low-dose dexamethasone or prednisone appears reasonable in patients with symptomatic hormone-refractory prostate cancer who are averse to receiving chemotherapy.

### ESTROGENS

Estrogens were used originally as therapy for hormone-sensitive prostate cancer and provided similar efficacy to orchiectomy. However, they increase the risk of thromboembolic events and for this reason estrogens are now used rarely in the treatment of hormone-sensitive disease. More recently, there is evidence that some patients with ‘hormone-resistant’ disease (i.e. after primary androgen ablation and addition and withdrawal of a peripheral antiandrogen) may have transient responses to estrogens such as diethylstilbestrol (Farrugia *et al*, 2000; Orlando *et al*, 2000). The mechanism is unknown, but this effect might also account for responses to the agent PC-SPES, which contains phytoestrogens, that have been reported for patients with ‘hormone-resistant’ disease (Small *et al*, 2000a). In view of the potential toxicity of estrogens in elderly men, they are recommended for use only in selected patients who have progressed after other types of treatment.

### MITOXANTRONE AND PREDNISONE

Since the goal of treatment was relief of symptoms, a Canadian group used ‘palliative response’ as the primary end point for their study of mitoxantrone and prednisone chemotherapy for patients with hormone-refractory prostate cancer (Tannock *et al*, 1996). In all, 161 patients from 11 Canadian institutions were randomised to receive mitoxantrone (12 mg m^−2^ every 3 weeks intravenously (i.v.)) plus prednisone 5 mg twice per day or prednisone alone. As assessed by either a stringent reduction in pain without increase in the level of analgesic medication or by a 50% reduction in analgesic medication without increase in pain, the palliative response rate was greater for patients receiving chemotherapy (38 *vs* 21%, *P*=0.025). Duration of symptomatic response was also longer for those on chemotherapy and patients who met the criterion for palliative response had improvements in most domains of quality of life, including improvement in overall sense of well being (Osoba *et al*, 1999). There was an imperfect correlation between PSA response and palliative response (Dowling *et al*, 2001). These results are consistent with those of a study by the Cancer and Leukaemia Group B (CALGB), which showed improved PSA response and pain control with mitoxantrone and hydrocortisone as compared to hydrocortisone alone (Kantoff *et al*, 1999). A more recent randomised trial by the Canadian Group has shown a consistent rate of pain response of 39% to mitoxantrone and prednisone among 209 patients with pain using similar criteria, and a 29% PSA response rate, but no significant benefit from adding the bisphosphonate, clodronate (Ernst *et al*, 2003). Mitoxantrone and prednisone are very well tolerated and can be given to elderly patients. Nausea, vomiting and alopecia are rare, and myelosuppresion is predictable and rarely a problem. However, the total dose is limited to about 120 mg m^−2^ because of cardiotoxicity at higher doses.

### TAXANES AND ESTRAMUSTINE

Docetaxel and paclitaxel are cytotoxic agents that act by stabilising microtubules, thereby disrupting the cellular microtubule network that is integral to cell division. Estramustine contains both estrogenic and alkylating groups and has also been reported to depolymerise microtubule-associated proteins. Taxanes have been evaluated in several studies for activity against hormone-refractory prostate cancer either as single agents or in combination with estramustine. The rationale for combining taxanes and estramustine is to achieve greater inhibition of microtubule function and cytotoxicity by binding to different protein targets in the same microtubular system.

Phase II trials in which patients with hormone-refractory prostate cancer have been treated with single-agent paclitaxel or docetaxel are summarised in Table 2

**Table 2:** Summary of phase II trials using a single-agent taxane in patients with hormone-refractory prostate cancer

**Author**	**Taxane regimen**	***N***	**Median age (yrs)**	**>50% PSA decline**	**Response in measurable disease**	**TTP**	**Survival**
Picus and Shultz (1999)	Docetaxel 75 mg m^−2^ q 3 wk	35	70	46%	28% (7/25)	9 m	27 m
Friedland *et al* (1999)	Docetaxel 75 mg m^−2^ q 3 wk	16	69	38%	66% (6/9)	NR	NR
Berry *et al* (2001)	Docetaxel 36 mg m^−2^ wk^−1^ × 6 of an 8-wk cycle	59	72	41%	33% (2/6)	5.1 m	9.4 m
Beer *et al* (2001)	Docetaxel 36 mg m^−2^ wk^−1^ × 6 of an 8-wk cycle	24	72	46%	40% (2/5)	NR	NR
Trivedi *et al* (2000)	Paclitaxel 150 mg m^−2^ wk^−1^	18	69	39%	50% (4/8)	NR	NR

*N*=number of patients; NR=not reported; PSA=prostate-specific antigen; q=every; TTP=time to progression; wk=week; yrs=years; q=every.

. The doses and scheduling of the taxanes vary between studies, but the end points of PSA response and response of measurable soft-tissue metastases were similar; the latter applies to only a minority of the patients enrolled on these studies. The range of PSA response rates of single-agent taxanes of 38–46% is greater than the PSA response rate in phase III studies of mitoxantrone and prednisone (∼30%). However, it can be misleading to make such comparisons as response rates often fall when an agent moves from the phase II to the phase III setting. One of the few studies to assess palliative response was conducted by Beer *et al* (2001). In this study, palliative response was seen in 48% of patients and PSA response was achieved in 46% of evaluable patients. Neutropenia was the predominant toxicity in all taxane studies. However, taxanes may also cause neuropathy, changes in the joints, skin and nails, and fatigue. The use of weekly taxanes in elderly patients with various tumour types has demonstrated a more favourable toxicity profile than an every-3 week regimen, while maintaining comparable levels of antitumour activity.

Phase II clinical trials in which a taxane was combined with estramustine are summarised in Table 3

**Table 3:** Summary of phase II clinical trials of a taxane plus estramustine for patients with hormone-refractory prostate cancer

**Author**	**Taxane regimen**	***N***	**Median age (yrs)**	**>50% PSA decline**	**Response in measurable disease**
Hudes *et al* (1997)	Paclitaxel 120 mg m^−2^ q 3 wk	34	69	53%	44% (4/9)
Smith *et al* (1999)	Paclitaxel 135 mg m^−2^ q 3 wk	37	64	65%	45% (10/22)
Petrylak *et al* (2000)	Docetaxel 70 mg m^−2^ q 3 wk	37	69	68%	55% (4/7)
Savarese *et al* CALGB (2001)	Docetaxel 70 mg m^−2^ q 3 wk+hydrocortisone	46	73	68%	50% (12/24)
Sinibaldi *et al* (2002)	Docetaxel 70 mg m^−2^ q 3 wk	40	68	45%	20% (4/20)
Copur *et al* (2001)	Docetaxel 30 mg m^−2^ wk	30	74	76%	58%

CALGB=Cancer and Leukaemia Group B; hr=hour; *N*=number of patients; PSA=prostate-specific antigen; wk=week; yrs=years; q=every; d=day.

. The rates of PSA response in these studies were 45–68%. The risk of thrombosis with a combination of a taxane and estramustine was higher than reported for either agent alone and justified the use of anticoagulation for prophylaxis of thromboembolic events. It is not clear whether estramustine is truly enhancing the activity of the taxane in these trial, or simply adding the season's responses that may sometimes be seen with estrogens in this population.

The high PSA response rates observed in taxane-based studies have led to trials comparing these regimens to the standard of mitoxantrone and prednisone. In a small randomised study, 130 patients with hormone-refractory prostate cancer received either of two doses of docetaxel plus estramustine and prednisone or mitoxantrone and prednisone. Preliminary data suggest a higher rate of PSA response, and greater clinical benefit, with docetaxel and estramustine, but this trial is small and has been published only in abstract form (Oudard *et al*, 2002).

Two large phase III studies comparing mitoxantrone and prednisone to docetaxel-based regimens have recently completed accrual. In the international TAX 327 study, more than 1000 patients were randomised to docetaxel plus prednisone given as either a 3-weekly or weekly regimen or to mitoxantrone plus prednisone. End points will include overall survival, PSA response and palliative response in patients with pain. In the SWOG 9916 study, more than 650 patients with hormone-refractory prostate cancer were randomised to estramustine and docetaxel or to mitoxantrone and prednisone. Again the primary end point is overall survival. Owing to greater toxicity, taxanes should only replace mitoxantrone as preferred treatment if they have a substantial effect to improve survival.

In Scenario 3, Mr Smith would have a reasonable probability of benefit from chemotherapy to improve his symptoms and quality of life. Mitoxantrone and prednisone are well tolerated and remains the current standard. There are no data to suggest that Mr Smith would obtain survival benefit from the introduction of chemotherapy.

## CONCLUSIONS

Numerous studies are underway to assess the role of chemotherapy for patients with various stages of prostate cancer. Mitoxantrone and prednisone have been shown to provide palliative benefit to about 30–40% of patients with symptoms. Results of phase III studies, which compare this current standard to taxane chemotherapy for patients with advanced metastatic disease will become available in 2004. However, even if taxanes with prednisone or estramustine show an effect to improve survival, their greater toxicity will require selection of patients with better performance status to receive them. Use of chemotherapy in the adjuvant or neoadjuvant setting is experimental and should only be undertaken in the context of a well-designed clinical trial.
